# A Lightweight Temporal–Spatial Fusion Network for Neonatal Sleep Staging

**DOI:** 10.3390/bioengineering13070723

**Published:** 2026-06-24

**Authors:** Ligang Zhou, Laishuan Wang, Yan Xu, Chen Chen

**Affiliations:** 1 Center for Medical Research and Innovation, Shanghai Pudong Hospital, Human Phenome Institute, Fudan University, 825 Zhangheng Road, Pudong, Shanghai 201203, China; 21110720086@m.fudan.edu.cn; 2 Department of Neurology, National Children’s Medical Center, Children’s Hospital of Fudan University, 399, Wangyuan Road, Minhang, Shanghai 201102, China; laishuanwang@fudan.edu.cn

**Keywords:** EEG, deep learning, lightweight, neonate, sleep staging, temporal–spatial

## Abstract

Background: Accurate assessment of neonatal sleep is critical for monitoring brain development and identifying potential neurological disorders, yet manual scoring of multi-channel EEG recordings is labor-intensive and prone to variability. Methods: To address this, we propose a lightweight temporal–spatial feature fusion network for automatic neonatal sleep staging. The model employs a dual-branch architecture to separately capture temporal dependencies and spatial correlations in EEG signals, which are then integrated through feature concatenation and a compact classifier to obtain comprehensive feature representations while maintaining low computational complexity. Results: The framework was evaluated on a clinical neonatal dataset (CHFD) for tasks including sleep–wake classification, quiet sleep detection, and three-stage sleep staging, achieving superior performance compared with several state-of-the-art methods. Additional evaluation on the MASS-S3 adult dataset demonstrate that the model retains competitive accuracy and F1-score, indicating strong generalization across populations. Conclusions: These results suggest that jointly modeling temporal and spatial features enables robust and efficient automatic sleep staging. The proposed approach offers a practical solution for clinical applications and edge deployment, providing reliable, multi-dimensional assessment of neonatal brain activity and laying the groundwork for future studies integrating larger datasets or multimodal physiological signals.

## 1. Introduction

Sleep plays a fundamental role in human physiological and neurological development, particularly during the neonatal period. In early life, sleep is closely associated with brain maturation, synaptic plasticity, and cognitive development, and abnormalities in neonatal sleep patterns may indicate neurological dysfunction or developmental disorders [1,2]. Continuous monitoring and analysis of neonatal sleep architecture, especially in neonatal intensive care units (NICUs), therefore provide critical insights into brain development and clinical outcomes [3]. Among various physiological signals, electroencephalography (EEG) is considered the most informative modality for characterizing neonatal sleep states due to its direct reflection of cortical activity. Traditionally, sleep staging is performed manually by trained experts using polysomnography (PSG), which remains the clinical gold standard [4,5]. However, manual scoring is labor-intensive, time-consuming, and subject to inter-rater variability. These limitations have motivated the development of automated sleep staging methods, particularly those based on EEG signals, to improve efficiency, consistency, and accessibility of sleep assessment [6].

Early studies on automated sleep staging primarily relied on traditional machine learning methods, which typically involved handcrafted feature extraction followed by classifiers such as support vector machines or random forests [7,8]. However, their reliance on manually designed features limits their ability to capture complex patterns in EEG signals. With the advancement of deep learning, data-driven methods have demonstrated superior capability in learning hierarchical representations directly from raw signals, significantly improving performance in sleep staging tasks. For example, DeepSleepNet employs deep CNNs to learn representative sleep features automatically [9]. Recurrent neural networks (RNNs), particularly long short-term memory (LSTM) networks, have been introduced to capture temporal dependencies across sleep epochs, as demonstrated in SeqSleepNet [10]. To further enhance feature representation, attention mechanisms have been incorporated to adaptively focus on informative temporal segments, while Transformer-based models, such as SleepTransformer, leverage self-attention to model long-range dependencies more effectively [11]. In addition, to explicitly model spatial relationships among EEG channels, graph neural networks (GNNs) have been explored, such as GraphSleepNet, which capture inter-channel connectivity patterns and improve representation learning [12].

However, the above methods either focus on a single aspect of EEG signals, such as temporal dynamics or single-channel representations, while neglecting the intrinsic spatial correlations among multiple EEG channels, or computationally intensive, involving large numbers of parameters and complex architectures, which restrict their deployment in resource-constrained environments such as bedside monitoring systems or wearable devices. On the other hand, neonatal EEG signals exhibit strong non-stationarity, low signal-to-noise ratio, and rapid developmental variability across subjects and ages, showing a higher inter-class similarity and ambiguity [13,14,15]. Most of the methods are tailored for adult sleep data, although these methods achieve strong performance on adult datasets, their direct application to neonatal EEG often results in significant performance degradation due to domain differences. Even for methods trained on limited neonatal datasets, their generalization ability across different populations remains insufficient, making them less reliable in real-world clinical scenarios. These challenges collectively highlight the need for models that are not only efficient and lightweight but also capable of capturing comprehensive temporal–spatial representations with strong generalization ability.

To address the above challenges, we propose a lightweight temporal–spatial fusion network for neonatal sleep staging based on multi-channel EEG signals. The proposed model adopts a dual-stream architecture to separately learn temporal dynamics and spatial correlations, and subsequently fuses these complementary representations to obtain a more comprehensive understanding of brain activity. By jointly modeling temporal and spatial information, the proposed approach enhances feature representation capability while maintaining a compact model size. In addition, the lightweight design significantly reduces computational overhead, making it suitable for deployment in edge computing scenarios. Unlike many existing neonatal sleep staging studies that focus exclusively on dataset construction or model development, this work simultaneously investigates both clinically meaningful neonatal EEG data collection and lightweight deep learning framework design. Extensive experiments demonstrate that the proposed method not only achieves competitive performance on neonatal datasets but also exhibits strong generalization ability across different populations, including adult sleep datasets.

In this work, we make two primary contributions. First, we construct and analyze a neonatal EEG sleep staging dataset collected from the Children’s Hospital of Fudan University (CHFD), which contains clinically annotated neonatal sleep recordings. Second, we propose a lightweight temporal–spatial fusion framework for automatic sleep staging. To better evaluate the generalization capability of the proposed method, additional experiments are conducted on the publicly available MASS-SS3 adult sleep dataset.

## 2. Materials and Methods

### 2.1. Dataset and Preprocessing

#### 2.1.1. Children’s Hospital of Fudan University Dataset

A clinical neonatal sleep dataset collected from the Children’s Hospital of Fudan University (CHFD) is employed to evaluate the proposed method. This dataset consists of 64 EEG recordings from neonates, with an average duration of approximately 131 min per recording. EEG data acquisition was performed using a Nicolet system, following the international 10–20 electrode placement scheme with channels F3, F4, C3, C4, T3, T4, P3, and P4, and a sampling rate of 500 Hz. The study protocol was approved by the ethics committee of CHFD (Approval No. (2017) 89). Based on established clinical guidelines for neonatal sleep staging, all recordings were manually annotated by an experienced neurophysiologist into three categories: wakefulness, quiet sleep (QS), and active sleep (AS) according to the practical guidelines and recommendations for neonatal sleep staging [16,17,18,19]. The detailed information of the neonatal sleep dataset is shown in Table 1.

The CHFD dataset is a private clinical dataset collected from the Children’s Hospital of Fudan University and is currently not publicly available due to ethical and privacy restrictions involving neonatal EEG recordings.

#### 2.1.2. MASS-SS3 Dataset

To further assess the generalization capability of the proposed method, additional experiments are conducted on adult sleep datasets. Specifically, a publicly available dataset, montreal archive of sleep studies (MASS), is adopted to evaluate the model in multi-channel sleep staging tasks on adult sleep data [20]. The MASS-SS3 dataset is selected because its EEG channel configuration is consistent with that of our private neonatal CHFD dataset, which allows for a fair and coherent cross-dataset evaluation under the same model architecture. In contrast, other widely used public datasets, such as Sleep-EDF and SHHS, either provide single-channel EEG recordings or employ different electrode configurations, making them less suitable for evaluation within a multi-channel framework [21,22]. Detailed information about the MASS-SS3 dataset is provided in Table 2.

The MASS-SS3 dataset was employed as an independent adult sleep staging benchmark to evaluate the cross-population generalization capability of the proposed framework. The MASS-SS3 dataset is selected because its EEG channel configuration is consistent with that of our private neonatal CHFD dataset, enabling a unified multi-channel input setting for model training and evaluation across datasets.

#### 2.1.3. Preprocessing

To enhance signal quality and ensure compatibility with the model input, the raw EEG recordings are first processed using a zero-phase notch filter to remove power-line interference. Specifically, for CHFD recordings, a 50 Hz notch filter was applied to remove power-line interference, while a 60 Hz notch filter was adopted for the MASS-SS3 dataset according to the corresponding recording environments. Using zero-phase filtering ensures that the phase of the EEG signals is preserved during the filtering process. Subsequently, a zero-phase band-pass filter with a frequency range of 0.3–35 Hz is applied. This frequency range is commonly adopted in sleep EEG studies, as it retains the key components of sleep-related brain activity, including delta (0.5–4 Hz), theta (4–8 Hz), alpha (8–13 Hz), and low beta (13–30 Hz) rhythms, while attenuating low-frequency drifts (below 0.3 Hz) caused by factors such as perspiration, electrode movement, or respiration, as well as high-frequency noise (above 35 Hz) mainly arising from muscle artifacts and external interference [23,24,25]. Zero-phase filtering is used to prevent distortion of the temporal characteristics of the EEG signals. After filtering, z-score normalization was independently applied to each EEG channel to reduce inter-subject amplitude variability and improve training stability. The EEG recordings are segmented into 30-s epochs, consistent with standard manual sleep scoring protocols. Epochs with substantial artifacts, interruptions, or abnormal patterns are manually discarded to ensure data quality. Finally, the EEG signals are downsampled from 500 Hz to 100 Hz to reduce computational load while retaining sufficient temporal resolution for effective analysis.

### 2.2. Temporal–Spatial Feature Fusion Network

**Figure 1:**
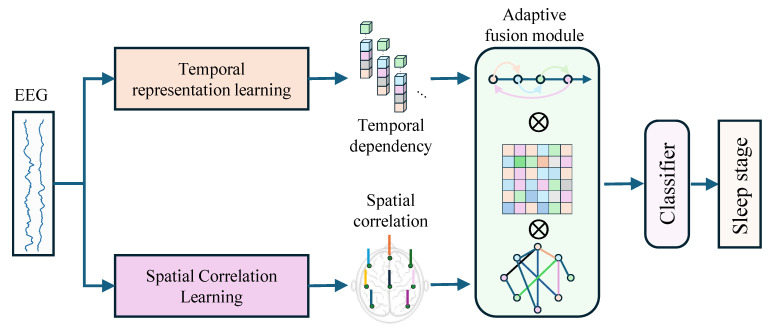
Architecture of the proposed method.

The overall architecture of the proposed framework is illustrated in Figure 1. The model follows a dual-branch design to jointly learn temporal dependencies and spatial correlations from multi-channel EEG signals. The input EEG segment is denoted as $X\in R^{B\times C\times L}$, where *B* is the batch size, C=8 is the number of EEG channels (F3, F4, C3, C4, T3, T4, P3, and P4), and L=3000 is the sample length of a 30 s epoch after downsampling to 100 Hz. The temporal branch extracts temporal representations from all channels, whereas the spatial branch first extracts channel-wise representations and then models correlations among channels through an adaptive adjacency mechanism. The two refined representations are flattened, concatenated, and fed into a lightweight fully connected classifier.

#### 2.2.1. Temporal Representation Learning Branch

**Figure 2:**
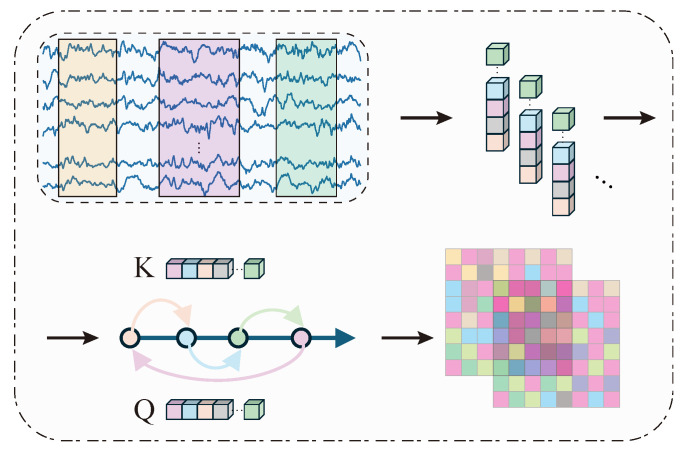
The illustration of the temporal representation learning branch.

The temporal representation learning branch is designed to extract local temporal patterns and model their dependencies, as shown in Figure 2.

Given an input EEG segment $X\in R^{B\times C\times L}$, where *B*, *C*, and *L* denote the batch size, number of channels, and the sample length of one segment, respectively, a lightweight convolutional neural network (CNN) is first applied along the temporal dimension to extract local features:

(1) $$F_{t}=\mathrm{CNN}\left(X\right)$$

where $F_{t}\in R^{B\times C\times T}$ represents the temporal feature embeddings.

To further capture dependencies among temporal features, a temporal attention mechanism is introduced. The features are projected into query and key spaces:

(2) $$Q=F_{t}^{⊤}W_{q},\;K=F_{t}^{⊤}W_{k}$$

where $W_{q},W_{k}\in R^{C\times H}$ are learnable projection matrices and *H* denotes the hidden dimension. $Q,K\in R^{B\times T\times H}$.

The dynamic dependency matrix is computed as

(3) $$A_{\mathrm{dyn}}=QK^{⊤}$$

To incorporate prior structural information, a learnable static adjacency matrix $A_{\mathrm{static}}\in R^{B\times T\times T}$ is introduced. The final temporal dependency matrix is defined as

(4) $$A_{t}=\sigma \left(A_{\mathrm{dyn}}+A_{\mathrm{static}}\right),\in R^{B\times T\times T}$$

where σ(·) denotes a non-linear activation function (ReLU). The resulting matrix $A_{t}\in R^{B\times T\times T}$ encodes the dependency relationships among temporal features.

For reproducibility, the temporal CNN is implemented as follows: Conv1d(8→32, kernel size = 50, stride = 2, padding = 25, bias = False), BatchNorm1d(32), ReLU; Conv1d(32→64, kernel size = 1, bias = False), BatchNorm1d(64), ReLU; Conv1d(64→64, kernel size = 16, stride = 2, padding = 8, bias = False), BatchNorm1d(64), ReLU; Dropout; and AdaptiveAvgPool1d(32). The output of this CNN is $F_{t}^{0}\in R^{B\times 64\times 32}$, which is transposed to $F_{t}\in R^{B\times T\times D_{t}}$ with T=32 and $D_{t}=64$ before adaptive learning.

The temporal dependency learner is implemented as an adaptive adjacency module with the sequence length of 32, the feature dimension of 64, and the hidden dimension of 64. Specifically, $F_{t}$ is projected into query and key embeddings, $Q_{t},K_{t}\in R^{B\times T\times H_{t}}$, where $H_{t}=64$. The dynamic temporal adjacency matrix is computed as $A_{\mathrm{dyn}}^{\left(t\right)}=Q_{t}K_{t}^{⊤}\in R^{B\times T\times T}$. A learnable static adjacency matrix $A_{\mathrm{static}}^{\left(t\right)}\in R^{T\times T}$ is broadcast to the batch dimension and combined with the dynamic matrix as $A_{t}=\sigma \left(A_{\mathrm{dyn}}^{\left(t\right)}+A_{\mathrm{static}}^{\left(t\right)}\right)$, where σ(·) denotes the ReLU activation. The refined temporal representation is obtained by matrix multiplication along the temporal dimension $F_{t}^{\prime }=A_{t}F_{t}\in R^{B\times 32\times 64}$ and is then flattened into a vector.

#### 2.2.2. Spatial Correlation Learning Branch

**Figure 3:**
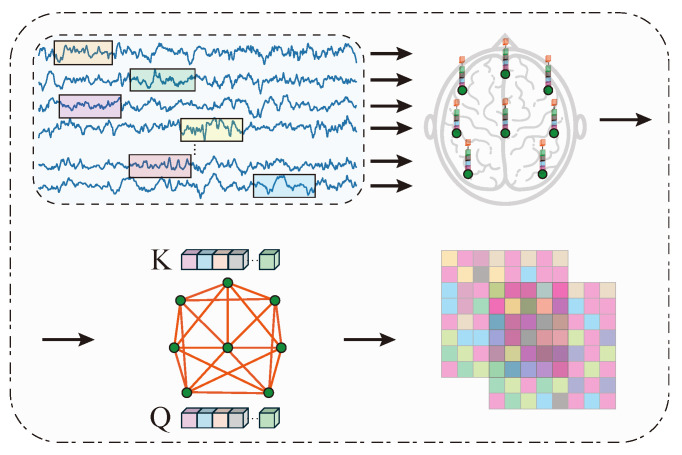
The illustration of the spatial correlation learning branch.

The spatial correlation learning branch focuses on modeling inter-channel relationships while preserving the spatial structure of EEG signals as shown in Figure 3.

The first part of this branch uses grouped one-dimensional convolution with G=C=8 groups. Therefore, each EEG channel forms an independent group and is processed separately. Importantly, this operation is not a convolution over an arbitrarily reordered one-dimensional channel sequence; instead, it is a depthwise temporal convolution applied independently to each electrode channel. This design avoids imposing an artificial neighborhood order among electrodes on the two-dimensional scalp surface.

(5) $$F_{s}=\mathrm{GroupCNN}\left(X\right)$$

where $F_{s}\in R^{B\times C\times S}$ denotes the channel independent feature embeddings. Specifically, the Group-CNN adopts a grouped convolution strategy [26,27], in which the input channels are divided into multiple groups and convolution is performed independently within each group.

To capture spatial correlations among channels, a spatial attention mechanism is employed. The features are projected into query and key representations:

(6) $$Q_{s}=F_{s}W_{q},\;K_{s}=F_{s}W_{k}$$

where $W_{q},W_{k}\in R^{S\times H}$ are learnable projection matrices and *H* denotes the hidden dimension. $Q_{s},K_{s}\in R^{B\times C\times H}$.

The dynamic spatial adjacency matrix is computed as

(7) $$A_{\mathrm{dyn}}^{\left(s\right)}=Q_{s}K_{s}^{⊤},\in R^{B\times C\times C}$$

Similarly, a learnable static adjacency matrix $A_{\mathrm{static}}^{\left(s\right)}\in R^{B\times C\times C}$ is introduced. The final spatial correlation matrix is given by

(8) $$A_{s}=\sigma \left(A_{\mathrm{dyn}}^{\left(s\right)}+A_{\mathrm{static}}^{\left(s\right)}\right)$$

where σ(·) denotes a non-linear activation function (ReLU).

The learned adjacency matrices are further utilized to refine feature representations before feature fusion:

(9) $$F_{t}^{\prime }=F_{t}A_{t},\in R^{B\times C\times T}$$

(10) $$F_{s}^{\prime }=F_{s}A_{s},\in R^{B\times C\times S}$$

The refined features are then fed into the feature fusion module.

The spatial CNN is implemented as follows: Conv1d(8→8, groups = 8, kernel size = 50, stride = 2, padding = 25, bias = False), BatchNorm1d(8), ReLU; Conv1d(8→8, groups = 8, kernel size = 1, bias = False), BatchNorm1d(8), ReLU; Conv1d(8→8, groups = 8, kernel size = 16, stride = 2, padding = 8, bias = False), BatchNorm1d(8), ReLU; Dropout; and AdaptiveAvgPool1d(256). The output is $F_{s}\in R^{B\times C\times S}$, where C=8 and S=256. Here, *S* denotes the dimensionality of the learned channel-wise spatial feature embedding.

After channel-wise feature extraction, correlations among EEG channels are modeled by the spatial adaptive adjacency module rather than by the grouped convolution itself. This module is implemented with seq_len = 8, feature_dim = 256, and hidden_dim = 256. Specifically, $F_{s}$ is projected into $Q_{s},K_{s}\in R^{B\times C\times H_{s}}$, where $H_{s}=256$, and the dynamic spatial adjacency matrix is calculated as $A_{\mathrm{dyn}}^{\left(s\right)}=Q_{s}K_{s}^{⊤}\in R^{B\times C\times C}$. A learnable static adjacency matrix $A_{\mathrm{static}}^{\left(s\right)}\in R^{C\times C}$ is then combined with the dynamic matrix to obtain $A_{s}=\sigma \left(A_{\mathrm{dyn}}^{\left(s\right)}+A_{\mathrm{static}}^{\left(s\right)}\right)$. Finally, the spatial representation is refined by $F_{s}^{\prime }=A_{s}F_{s}\in R^{B\times 8\times 256}$, so that correlations among channels are implicitly integrated into the spatial features before fusion. The flattened spatial representation is also 2048-dimensional.

#### 2.2.3. Adaptive Feature Fusion and Classification

To effectively integrate temporal and spatial features, an adaptive fusion module is proposed. This module first learns the relative importance of temporal and spatial representations using two sets of learnable weights. Given temporal features $F_{t}^{\prime }$ and spatial features $F_{s}^{\prime }$, element-wise (Hadamard) multiplication is applied:

(11) $${\tilde{F}}_{t}=W_{t}⊙F_{t}^{\prime },\;{\tilde{F}}_{s}=W_{s}⊙F_{s}^{\prime }$$

where $W_{t}$ and $W_{s}$ are learnable parameters, and ⊙ denotes element-wise multiplication.

The weighted features and the $F_{t}$ and $F_{s}$ are then flattened and projected into a unified feature space:

(12) $$Z_{t}=Flatten\left({\tilde{F}}_{t}\right)W_{t}^{\prime },\in R^{B\times D},\;Z_{s}=Flatten\left({\tilde{F}}_{s}\right)W_{s}^{\prime },\in R^{B\times D}$$

(13) $$F_{t}^{\prime \prime }=Flatten\left(F_{t}\right)W_{t}^{\prime \prime },\in R^{B\times D},\;F_{s}^{\prime \prime }=Flatten\left(F_{s}\right)W_{s}^{\prime \prime },\in R^{B\times D}$$

where, $W_{t}^{\prime }$, $W_{s}^{\prime }$, $W_{t}^{\prime }$, and $W_{s}^{\prime }$ denote learnable linear projection matrices that map temporal and spatial features into a unified latent embedding space. *D* represents the latent embedding dimension after global pooling and linear projection.

The projected features are integrated to form the final representation:

(14) $$Z=\mathrm{Concat}(Z_{t}+F_{t}^{\prime \prime },Z_{s}+F_{s}^{\prime \prime })$$

Finally, the fused representation is passed through a classifier composed of two fully connected layers. The output logits generated by FC2 are further normalized using the softmax function to obtain the final sleep stage probability distribution:

(15) $$logit=softmax(\sigma \left({\mathrm{FC}}_{2}\left(\sigma \left({\mathrm{FC}}_{1}\left(Z\right)\right)\right)\right))$$

(16) $$\hat{y}=Argmax\left(logit\right)$$

where σ(·) represents the ReLU activation function and $\hat{y}$ denotes the predicted sleep stage.

The model is optimized using the categorical cross-entropy loss function:

(17) $$L=-\sum_{i=1}^{N}y_{i}\log\left({logit}_{i}\right),$$

where $y_{i}$ and ${logit}_{i}$ denote the ground-truth label and predicted probability, respectively, and *N* represents the number of sleep-stage categories.

### 2.3. Evaluation Metrics

We evaluate the proposed approach using multiple performance metrics, including accuracy, macro-F1 score, Cohen’s kappa coefficient, macro-sensitivity, and macro-specificity. All experiments were performed using subject-wise 10-fold cross-validation to avoid subject leakage. Specifically, subjects were divided into 10 mutually exclusive folds; in each iteration, 9 folds were used for training and the remaining fold was used for testing. Each subject therefore appeared in the test set exactly once.

The proposed model was implemented in PyTorch version 2.11.0 and optimized using the Adam optimizer. The training hyperparameters were fixed across all datasets and tasks as follows: batch size = 64, initial learning rate = $1\times 10^{-3}$, weight decay = $1\times 10^{-5}$, dropout rate = 0.5, and training epochs = 150. No dataset-specific fine-tuning strategy was introduced for either CHFD or MASS-SS3; the same model architecture, training procedure, and validation protocol were used to ensure fair comparison and reproducibility. All experiments were conducted on a server equipped with an NVIDIA GeForce RTX 4090 GPU and an Intel Xeon Platinum 8383C CPU.

After completing the 10 folds, the predictions obtained from the test subjects of all folds were concatenated to reconstruct one complete out-of-sample prediction set for the entire dataset. The overall confusion matrix was then calculated from this aggregated prediction set, and all reported metrics in Table 3, Table 4 and Table 5 were derived from this overall confusion matrix. Therefore, the reported values represent dataset-level performance under subject-wise cross-validation rather than the arithmetic mean of fold-wise scores. Since the folds contain different subjects and different epoch distributions, reporting fold-wise standard deviations or confidence intervals would not directly represent repeated measurements on the same statistical samples. We therefore report the aggregated dataset-level metrics, which better reflect the model’s overall performance while still preserving subject-wise independence during testing.

## 3. Results

**Figure 4:**
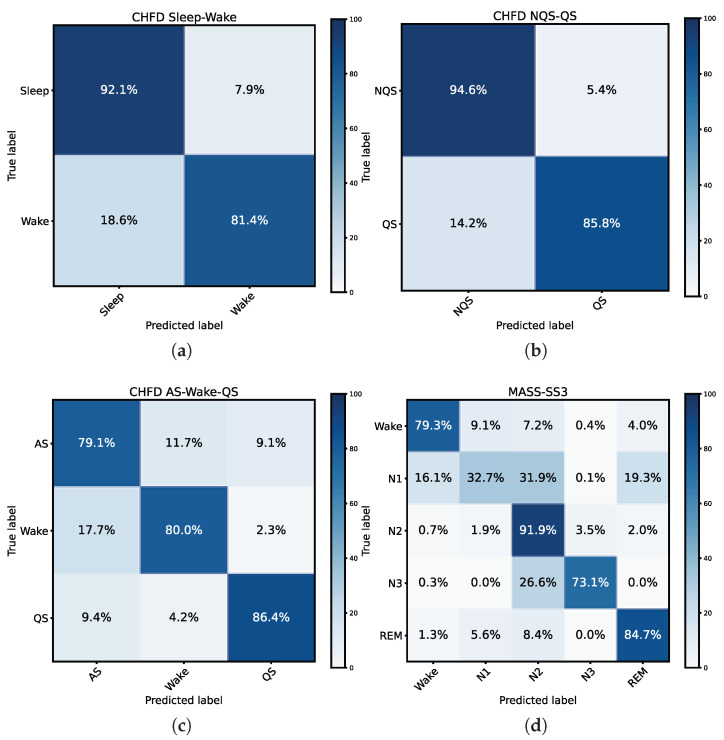
Confusion matrix of the proposed method on different sleep analysis tasks. (**a**) Confusion matrix on CHFD Sleep–Wake classification task. (**b**) Confusion matrix on CHFD QS detection task. (**c**) Confusion matrix on CHFD AS–Wake–QS classification task. (**d**) Confusion matrix on MASS-SS3 five-stage classification task. Confusion matrix obtained from the aggregated predictions of all test subjects across the 10-fold cross-validation procedure.

Comprehensive experiments are carried out to evaluate the performance of the proposed temporal–spatial feature fusion network for neonatal sleep staging. Our primary experiments focus on the CHFD dataset, which contains multi-channel EEG recordings from neonates, to assess the performance of the model in clinically relevant sleep staging tasks. To further evaluate the generalization capability of the proposed approach across populations, additional experiments are conducted on the MASS-SS3 adult sleep dataset. This additional validation allows us to examine whether the same temporal–spatial modeling strategy remains effective on adult multi-channel EEG under a unified architecture and training protocol, thereby assessing its robustness and applicability across different populations. The evaluation considers multiple performance metrics, including accuracy, F1-score, Cohen’s kappa coefficient, specificity, and sensitivity, providing a comprehensive assessment of the model’s discriminative power and reliability. In addition, to contextualize the performance of the proposed method, we conduct comparative experiments with several representative baseline approaches on the CHFD dataset, including both traditional machine learning methods and recent deep learning models. These comparisons allow us to highlight the advantages of our dual-branch temporal–spatial learning framework over existing approaches in neonatal sleep staging. The results are shown in Table 3 and Figure 4.

### 3.1. Performance on Sleep–Wake Task on CHFD Dataset

For the binary sleep–wake classification on the CHFD dataset, the proposed temporal–spatial feature fusion network achieved an overall accuracy of 88.6%, with an F1-score of 0.870 and a Cohen’s kappa of 0.740. The macro-averaged sensitivity and specificity were both 0.868, indicating balanced performance across the two classes. As shown in the corresponding confusion matrix, the model correctly identified 92.1% of sleep epochs and 81.4% of wake epochs. Misclassification primarily occurred when brief wake periods were embedded within sleep, which is consistent with the known challenges of distinguishing transient arousals in neonatal EEG. These results demonstrate the model’s robust capability to discriminate between sleep and wake states in neonates.

### 3.2. Performance on QS Detection Task on CHFD Dataset

For quiet sleep (QS) detection, the model achieved higher performance, with an overall accuracy of 91.6%, an F1-score of 0.906, and a kappa coefficient of 0.811. The macro-sensitivity and macro-specificity were both approximately 0.902. The confusion matrix indicates that 94.6% of non-quiet sleep (NQS) epochs and 85.8% of QS epochs were correctly classified. The slightly lower QS sensitivity reflects occasional confusion with non-quiet sleep stages, particularly during transitional periods. Overall, the results highlight the model’s effectiveness in detecting QS epochs, which is critical for assessing neonatal sleep architecture.

### 3.3. Performance on AS-W-QS Task on CHFD Dataset

For the three-class classification task involving active sleep (AS), wake (W), and quiet sleep (QS), the model achieved an overall accuracy of 81.9%, an F1-score of 0.819, and a kappa of 0.729. The macro-sensitivity and macro-specificity were 0.818 and 0.910, respectively, indicating strong discriminative capability across all three classes. From the confusion matrix, AS epochs were correctly classified with 79.1% accuracy, wake epochs with 80.0%, and QS epochs with 86.4%. Misclassifications predominantly occurred between AS and wake, reflecting the intrinsic similarity of EEG patterns during brief arousals. These results confirm that the proposed dual-branch temporal–spatial framework effectively captures both temporal dependencies and spatial correlations to distinguish subtle differences among neonatal sleep states.

### 3.4. Performance on Five-Stage Classification Task on MASS-SS3 Dataset

To evaluate the cross-population generalization capability of the proposed framework, experiments were further conducted on the public MASS-SS3 dataset for five-stage sleep classification (Wake–N1–N2–N3–REM). The proposed model achieved an overall accuracy of 82.0%, an MF1 score of 0.739, a Cohen’s kappa of 0.729, a macro-sensitivity of 0.723, and a macro-specificity of 0.944. According to the confusion matrix, N2 and REM stages were recognized with relatively high accuracy (91.9% and 84.7%, respectively), while N1 and N3 remained the most challenging stages due to their transitional and heterogeneous characteristics.

Table 5 compares the proposed method with several representative sleep staging approaches on the MASS-SS3 dataset. Although methods specifically designed and optimized for adult sleep staging, such as DeepSleepNet and GraphSleepNet, achieved higher overall performance, the proposed framework still obtained comparable results, achieving an accuracy of 82.0% and maintaining a high macro-specificity of 0.944. Considering that the model was originally developed for neonatal sleep staging, these results demonstrate its ability to generalize across different age groups and sleep scoring systems.

The promising performance on both neonatal and adult datasets suggests that the proposed temporal–spatial fusion framework captures sleep-related EEG representations that are not restricted to neonatal characteristics. Specifically, the temporal branch learns stage-dependent temporal dynamics, while the spatial branch models inter-channel relationships. By integrating complementary information from both perspectives, the proposed architecture exhibits robust cross-population applicability and good generalization capability.

### 3.5. Cross-Task Analysis

A joint analysis across all tasks provides deeper insights into the performance and robustness of the proposed temporal–spatial feature fusion network. On the CHFD dataset, the model demonstrates consistently high accuracy for both binary (Sleep–Wake) and QS detection tasks, with slightly lower performance observed in the three-class AS–W–QS task. This trend indicates that while the model effectively captures clear distinctions between sleep and wake or between QS and non-QS epochs, differentiating among closely related neonatal sleep states, particularly AS and wake, remains inherently challenging due to the subtle and transient EEG patterns during these transitions.

Comparing neonatal tasks with the adult five-stage classification task on MASS-SS3 highlights the generalization capability of the proposed framework. Despite differences in EEG characteristics between neonates and adults, the model achieves competitive performance across all five adult sleep stages, with particularly high accuracy in N2 and REM stages. Misclassifications primarily occur in N1 and N3 stages, reflecting the well-known ambiguity of these transitional and deep sleep stages.

Furthermore, the cross-dataset experimental results indicate that the proposed framework maintains stable classification capability even under substantial physiological differences between neonatal and adult EEG signals. Compared with neonatal EEG, adult EEG exhibits more mature sleep organization and more stable spectral characteristics. Nevertheless, the proposed model preserves robust performance, suggesting that the learned temporal–spatial representations possess strong generalization ability rather than overfitting to neonatal-specific EEG patterns.

The additional validation on the public MASS-SS3 dataset also strengthens the evidence that the proposed method is broadly applicable to different sleep staging tasks and populations. These findings support the claim that the proposed architecture can serve as a generalized EEG sleep staging framework beyond the neonatal domain.

These observations suggest that the dual-branch temporal–spatial architecture effectively captures generalized representations of sleep-related brain activity, enabling the model to maintain robust performance across multiple tasks and populations.

Additionally, the macro-sensitivity and macro-specificity values across tasks indicate balanced performance, with minimal bias toward any particular class. Overall, the cross-task analysis underscores the effectiveness of integrating temporal dependencies and spatial correlations, demonstrating that the proposed model can handle both neonatal and adult sleep staging tasks with high reliability, while providing a unified framework for multi-class sleep classification.

### 3.6. Comparison with Existing Methods

To further validate the effectiveness of the proposed method, a comparative study with several representative existing approaches was conducted on the CHFD dataset for the three-stage (AS–W–QS) classification task. The compared methods include both traditional machine learning-based approaches and advanced deep learning models, covering CNN, RNN, attention, and graph-based architectures [9,12,28,29,30,31,32,33]. All methods were evaluated under the same experimental settings using subject-wise 10-fold cross-validation to ensure fairness.

As shown in Table 4, the proposed method achieves the highest accuracy of 81.9% with an F1-score of 0.819, and a Cohen’s kappa of 0.729. In particular, the proposed model significantly outperforms classical CNN-based methods such as MB-CNN and Conv-2d, which rely heavily on handcrafted features or limited spatial modeling capability. For instance, MB-CNN achieves an accuracy of 72.8%, while Conv-2d methods exhibit even lower performance (approximately 52–53%), indicating that relying solely on local convolutional features is insufficient to capture the complex temporal–spatial patterns in neonatal EEG signals.

Compared with more advanced deep learning methods, such as DeepSleepNet and AttnSleep, the proposed method also demonstrates clear advantages [9,31]. Although DeepSleepNet leverages multi-scale CNN and bi-directional LSTM to model temporal dependencies, its performance (68.9% accuracy) is limited, likely due to its focus on single-channel input and insufficient modeling of inter-channel relationships. Similarly, AttnSleep introduces attention mechanisms but still falls short (68.0% accuracy), suggesting that temporal attention alone is not sufficient without effective spatial modeling.

Graph-based approaches, such as GraphSleepNet and MVST-GCN, attempt to capture spatial relationships among EEG channels [12,33]. However, their performance remains suboptimal (68.9% and 69.7% accuracy, respectively), which may be attributed to their reliance on handcrafted features or fixed graph structures that limit their adaptability. In contrast, the proposed method introduces an adaptive adjacency learning mechanism that jointly considers both static and dynamic relationships, enabling more flexible and data-driven modeling of channel interactions.

Among all compared methods, MS-HNN achieves relatively competitive performance (75.4% accuracy), as it incorporates both temporal learning and multi-scale feature extraction tailored for neonatal data. Nevertheless, the proposed method still surpasses MS-HNN by a notable margin across all evaluation metrics. This improvement can be attributed to the proposed dual-branch architecture, which explicitly disentangles temporal and spatial feature learning and further integrates them through feature concatenation and a compact classifier.

In addition to performance improvements, the proposed method maintains a favorable trade-off between accuracy and model complexity. With only 0.81 M parameters, it is significantly more lightweight than models such as DeepSleepNet (24.75 M) and MS-HNN (25.63 M), while achieving superior or comparable results. Although some lightweight models (e.g., MB-CNN, GraphSleepNet) have fewer parameters, their performance is considerably lower, highlighting the effectiveness of the proposed design in balancing efficiency and accuracy. Meanwhile, inference efficiency was also evaluated. The proposed lightweight framework achieves low computational complexity and reduced inference time, demonstrating its suitability for edge deployment and real-time neonatal sleep monitoring applications.

Overall, these results demonstrate that the proposed temporal–spatial feature fusion network effectively leverages multi-channel EEG information, capturing both temporal dependencies and spatial correlations in a unified framework. This leads to superior performance compared to existing methods, while maintaining a lightweight architecture suitable for practical deployment.

## 4. Discussion

In this study, we proposed a lightweight temporal–spatial feature fusion network for neonatal sleep staging and evaluated its performance on both neonatal and adult EEG datasets. The results demonstrate that the model consistently achieves high accuracy, F1-score, and Cohen’s kappa across multiple classification tasks, including binary sleep–wake discrimination, quiet sleep (QS) detection, and three-stage AS–W–QS classification in neonates, as well as five-stage adult sleep classification. Compared with several representative state-of-the-art methods, our approach exhibits superior performance while maintaining a compact model size, highlighting the effectiveness of integrating temporal and spatial feature learning within a unified framework.

The strong performance on neonatal tasks suggests that explicitly modeling both temporal dependencies and spatial correlations is critical for capturing the complex dynamics of neonatal EEG signals. In the binary sleep–wake and QS detection tasks, the high sensitivity and specificity indicate that the model is able to accurately differentiate distinct brain states even in the presence of transient arousals or subtle EEG patterns. For the three-stage AS–W–QS classification, slightly lower accuracy reflects the inherent difficulty in distinguishing active sleep from wake periods, consistent with previous observations that AS and wake exhibit overlapping EEG features. The use of adaptive adjacency matrices, combining static and dynamic components, likely contributed to capturing subtle temporal and inter-channel interactions that traditional CNN or RNN methods fail to fully exploit.

The evaluation on the adult MASS-SS3 dataset provides additional evidence regarding the applicability of the proposed temporal–spatial framework beyond the neonatal cohort. The architecture was primarily designed for neonatal sleep staging, but the same architecture and training/validation protocol were applied to the adult MASS-SS3 dataset without dataset-specific fine-tuning. Differences in performance between CHFD and MASS-SS3 are expected because the two datasets differ substantially in acquisition settings, recording environments, scoring systems, target populations, sleep architecture, and EEG characteristics. In particular, neonatal EEG exhibits immature cortical organization and distinct AS/QS patterns, whereas adult sleep staging follows the W–N1–N2–N3–REM structure. Therefore, the MASS-SS3 experiment is intended to verify the effectiveness and robustness of the proposed temporal–spatial modeling strategy on adult multi-channel EEG, rather than to claim that a neonatal-oriented model is fully optimized for adult sleep staging.

From a physiological perspective, neonatal EEG differs substantially from adult EEG in terms of spectral composition and sleep organization. Neonatal EEG signals exhibit higher discontinuity, immature cortical synchronization, and evolving sleep architecture. For example, neonatal quiet sleep is typically characterized by discontinuous delta-dominant activity, whereas adult N3 sleep exhibits more continuous and stable slow-wave activity. Similarly, neonatal active sleep differs from adult REM sleep in both EEG amplitude and temporal organization.

These domain differences explain why performance variations across CHFD and MASS-SS3 are normal. The proposed method does not rely on handcrafted features specific to a single dataset; instead, it strengthens temporal and spatial association learning to obtain more discriminative sleep-state representations. The public adult dataset was included as an additional validation cohort to examine whether the same temporal–spatial design remains effective under a different population and scoring system.

The confusion matrix on the MASS-SS3 dataset further indicates that most classification errors occur between N1 and N2 stages, which is consistent with previous adult sleep staging studies due to the transitional nature of these stages and their overlapping theta-wave characteristics. In contrast, N2 and REM stages achieve relatively high classification accuracy because of their more distinctive temporal and spectral EEG patterns.

Compared with existing methods, our model demonstrates clear advantages. The proposed framework is a nonlinear deep neural network architecture composed of convolutional feature extraction modules, adaptive dependency learning modules, temporal–spatial feature fusion, and fully connected classifiers, rather than a linear regression model. Traditional single-channel CNN-based methods, such as Conv-2d and MB-CNN, achieve limited performance, highlighting the importance of multi-channel spatial information. Graph-based and attention-based models (GraphSleepNet, MVST-GCN, AttnSleep) improve performance by incorporating spatial or temporal dependencies, but often rely on handcrafted features or fixed adjacency structures, limiting adaptability. In contrast, the proposed approach jointly models temporal and spatial features with adaptive, data-driven adjacency learning, which allows the network to dynamically capture complex interactions between channels and across time. Notably, while MS-HNN also targets neonatal sleep with multi-scale CNN and temporal learning, our dual-branch design with explicit temporal–spatial fusion further improves classification performance while maintaining a smaller parameter footprint, emphasizing the efficiency and practicality of the approach.

The findings have several implications for neonatal and clinical sleep research. First, accurate automated sleep staging enables continuous monitoring of neonatal brain development, which is critical for early detection of neurodevelopmental disorders and assessment of treatment effects. Second, the lightweight design and multi-channel integration suggest that the model can be feasibly deployed in bedside monitoring systems or wearable EEG devices, facilitating real-time and scalable neonatal sleep assessment. Third, the demonstrated generalization to adult datasets indicates the potential for broader applications in sleep research, including cross-population studies and investigations of sleep-related neurological conditions.

Several limitations should be acknowledged. First, despite the promising performance, the neonatal dataset remains relatively small and may not fully capture the diversity of EEG patterns across different gestational ages, developmental stages, or clinical conditions. Consequently, the generalizability of the proposed model could be further improved by training and validating on larger and more heterogeneous datasets. Second, although the proposed framework achieves a favorable balance between performance and model complexity, the relationship between network capacity and classification performance has not been systematically investigated. Future work should explore model scaling strategies, including reduced network widths and hidden dimensions, to identify potential parameter redundancy and determine the minimum model capacity required to maintain robust performance. Third, while the current feature concatenation and compact classifier provide an efficient temporal–spatial integration strategy, more advanced fusion mechanisms, such as graph attention networks or transformer-based cross-channel attention, may further enhance representation learning. Future studies may also focus on longitudinal analyses of developmental trajectories and on integrating additional physiological signals, such as ECG and respiratory activity, to enable multimodal neonatal sleep assessment [34]. Finally, interpretability analyses of the learned temporal–spatial dependencies could provide deeper insights into the neurophysiological mechanisms underlying neonatal sleep states and facilitate clinical translation of the proposed framework [35].

## 5. Conclusions

In this paper, we proposed a lightweight temporal–spatial feature fusion network for automatic neonatal sleep staging using multi-channel EEG signals. The model employs a dual-branch architecture to capture temporal dependencies and spatial correlations, and it integrates the refined temporal and spatial representations through feature concatenation and a compact fully connected classifier. Experimental results on the CHFD dataset demonstrate that the proposed method achieves superior performance across multiple neonatal sleep staging tasks compared with existing approaches. In addition, evaluation on the MASS-SS3 adult dataset shows that the same temporal–spatial modeling strategy remains effective on adult multi-channel EEG without dataset-specific fine-tuning, supporting its robustness across different populations. Overall, the proposed method provides an effective and efficient solution for neonatal sleep staging, with potential for practical deployment in real-world clinical scenarios. Future work will focus on improving generalization with larger datasets and exploring multimodal extensions.

## Figures and Tables

**Table 1:** Specifications of the CHFU dataset.

Terms	Details
Gender (b:g)	32:32
Gestational age (w + d)	38.3 ± 1.8
Postmenstrual age (w + d)	40.5 ± 1.7
Weight (kg)	3.3 ± 0.6
Number of wakefulness epochs	5514 (32.8%)
Number of QS epochs	5749 (34.2%)
Number of AS epochs	5540 (33.0%)
EEG channel	F3, F4, C3, C4, T3, T4, P3, and P4
Sampling rate	500 Hz

b:g means boy: girl; the notation “w + d” refers to gestational age measured in weeks and days. Specifically, “38.3 ± 1.8” indicates a mean gestational age of 38.3 weeks with a standard deviation of 1.8 weeks.

**Table 2:** Specifications of the MASS-SS3 dataset.

Terms	Details
Gender (m:f)	28:34
Scoring rules	AASM
Sampling rate	256 Hz
Number of wakefulness epochs	6442 (10.9%)
Number of N1 epochs	4839 (8.2%)
Number of N2 epochs	29,802 (50.2%)
Number of N3 epochs	7653 (12.9%)
Number of REM epochs	10,581 (17.8%)
Selected EEG channel	F3, F4, C3, C4, T3, T4, P3, and P4

m:f means male: female.

**Table 3:** Performance evaluation on CHFD and MASS-SS3 datasets.

Dataset	Task	Accuracy	MF1	Kappa	Macro-Sensitivity	Macro-Specificity
CHFD	Sleep–Wake	0.886	0.870	0.740	0.868	0.868
CHFD	QS Detection	0.916	0.906	0.811	0.902	0.902
CHFD	AS-W-QS	0.819	0.819	0.729	0.818	0.910
MASS-SS3	W-N1-N2-N3-REM	0.820	0.739	0.729	0.723	0.944

M-sens: macro-sensitivity, M-spec: macro-specificity. The reported metrics are calculated from the aggregated predictions of all test subjects across the 10 folds.

**Table 4:** Comparison of different methods on the CHFD AS-W-QS task.

Method	Accuracy	MF1	Kappa	M-Sens	M-Spec	Parameters
MB-CNN [28]	0.728	0.682	0.561	0.671	0.850	<0.01 M
Conv-2d [29]	0.535	0.531	0.489	0.768	0.536	<0.01 M
Conv-2d [30]	0.523	0.519	0.411	0.761	0.523	<0.01 M
DeepSleepNet [9]	0.689	0.682	0.535	0.845	0.692	24.75 M
AttnSleep [31]	0.680	0.646	0.659	0.839	0.650	5.20 M
MS-HNN [32]	0.754	0.758	0.728	0.876	0.755	25.63 M
GraphSleepNet [12]	0.689	0.682	0.535	0.845	0.692	<0.05 M
MVST-GCN [33]	0.697	0.696	0.547	0.849	0.699	<0.05 M
Proposed	0.819	0.819	0.729	0.818	0.910	0.81 M

M-sens: macro-sensitivity, M-spec: macro-specificity. The reported metrics are calculated from the aggregated predictions of all test subjects across the 10 folds.

**Table 5:** Comparison of different methods on the MASS-SS3 W-N1-N2-N3-REM task.

Method	Accuracy	MF1	Kappa	M-Sens	M-Spec
DeepSleepNet [9]	0.843	0.845	0.765	0.852	0.923
AttnSleep [31]	0.827	0.751	0.756	0.750	0.954
GraphSleepNet [12]	0.856	0.785	0.782	0.773	0.955
Proposed	0.820	0.739	0.729	0.723	0.944

M-sens: macro-sensitivity, M-spec: macro-specificity. The reported metrics are calculated from the aggregated predictions of all test subjects across the 10 folds.

## Data Availability

The datasets analyzed during the current study are not publicly available due to privacy and ethical restrictions involving neonatal patient data. Access to these clinical EEG recordings is restricted to authorized personnel. In the future, the authors may consider making de-identified data available for research purposes, pending appropriate approvals and ethical clearance.

## Abbreviations

The following abbreviations are used in this manuscript:

PSG	Polysomnography
NICUs	Neonatal intensive care units
CHFD	Children’s hospital of fudan dataset
MASS	Montreal archive of sleep studies
SHHS	Sleep heart health study
EEG	Electroencephalograph
CNN	Convolutional neural network
RNN	Recurrent neural network
GNN	Graph neural network
LSTM	Long short-term memory
QS	Quiet sleep
AS	Active sleep
